# Training Visual Imagery: Improvements of Metacognition, but not Imagery Strength

**DOI:** 10.3389/fpsyg.2012.00224

**Published:** 2012-07-10

**Authors:** Rosanne L. Rademaker, Joel Pearson

**Affiliations:** ^1^Cognitive Neuroscience Department, Maastricht UniversityMaastricht, Netherlands; ^2^Psychology Department, Vanderbilt UniversityNashville, TN, USA; ^3^Vanderbilt Vision Research Center, Vanderbilt UniversityNashville, TN, USA; ^4^School of Psychology, The University of New South WalesSydney, NSW, Australia

**Keywords:** visual imagery, training, learning, metacognition, introspection, binocular rivalry, consciousness

## Abstract

Visual imagery has been closely linked to brain mechanisms involved in perception. Can visual imagery, like visual perception, improve by means of training? Previous research has demonstrated that people can reliably evaluate the vividness of single episodes of imagination – might the metacognition of imagery also improve over the course of training? We had participants imagine colored Gabor patterns for an hour a day, over the course of five consecutive days, and again 2 weeks after training. Participants rated the subjective vividness and effort of their mental imagery on each trial. The influence of imagery on subsequent binocular rivalry dominance was taken as our measure of imagery strength. We found no overall effect of training on imagery strength. Training did, however, improve participant’s metacognition of imagery. Trial-by-trial ratings of vividness gained predictive power on subsequent rivalry dominance as a function of training. These data suggest that, while imagery strength might be immune to training in the current context, people’s metacognitive understanding of mental imagery can improve with practice.

## Introduction

Mental imagery can be described as the retrieval of perceptual information from memory, and the subsequent examination of this information in the “minds eye.” Research has provided a growing body of behavioral and neuroimaging evidence that there is considerable overlap between the “minds eye” and actual perception (Chen et al., 1998; Kreiman et al., 2000; O’Craven and Kanwisher, 2000; Zatorre and Halpern, 2005). For example, behavioral studies have demonstrated that imagery content can selectively influence perception (Perky, 1910; McDermott and Roediger, 1994; Pearson et al., 2008). Imagery has been shown to affect visual detection thresholds (Ishai and Sagi, 1997), performance on a visual acuity task (Craver-Lemley and Reeves, 1992), and to induce negative aftereffects (Gilden et al., 1995) in much the same way as a sensory stimulus. Recent neuroimaging studies show that there is considerable spatial overlap between activated areas of the brain during both visual perception and visual imagery, for example information about a pattern held in mind during working memory or imagery can be present in visual sensory cortex (Kosslyn et al., 1995; Slotnick et al., 2005; Harrison and Tong, 2009; Serences et al., 2009; Stokes et al., 2009). Like perception, visual imagery is impaired when visual cortical activity is disturbed by means of transcranial magnetic stimulation (Kosslyn et al., 1999).

If visual imagery can indeed be defined as the recreation of a perceptual representation in the absence of retinal input (Ishai and Sagi, 1995), one may wonder exactly how similar imagery is to perception. Specifically, prolonged visual practice can improve perceptual skill (Fahle and Poggio, 2002; Fine and Jacobs, 2002; Sasaki et al., 2010); can imagery also improve with daily practice? There is some evidence to suggest that perceptual learning can occur from training without physical stimulation. Repetitively imagining the crucial part of a visual bisection stimulus (visual spatial judgment) or imagining a low-contrast Gabor pattern (contrast judgment) can improve performance on subsequent perceptual tasks (Tartaglia et al., 2009). Similarly, imagining motor-acts facilitates performance on corresponding tasks by training relevant parts of motor cortex, and by strengthening associations between processes and actions (Driskell et al., 1994; Weiss et al., 1994; Feltz and Landers, 2007). To date, research has mainly focused on the effects imagery training has on subsequent perceptual tasks. Here, we look directly at the influence of imagery training on the strength of imagery itself.

One of the hallmarks of mental imagery is the considerable difference in reported imagery strength and vividness observed across individuals (Galton, 1883; McKellar, 1965; Marks, 1973; Amedi et al., 2005; Cui et al., 2007). Some individuals claim veridical, vivid imagery, while others doubt its entire existence (McKellar, 1965). The factors causing such differences in imagery strength remain largely unknown. One hypothesis is that individuals who actively practice, or whose everyday activities involve strong use of imagery, might have strengthened their imagery through training and practice (Sacks, 2010). We sought to examine such a proposal in the lab by engaging individuals in an imagery task daily, over a period of 5 days. Can repeated instances of forming visual imagery lead to improved imagery strength?

To address this question researchers must be able to reliably measure imagery strength from 1 day to the next. Previous work demonstrated that sustained imagery has a pronounced and visually specific impact on subsequent perception (Pearson et al., 2008, 2011). These studies utilized a visual phenomenon called binocular rivalry; when two different patterns are presented one to each eye, only one of the patterns is consciously perceived. Subtle experimental manipulations, such as attention (Meng and Tong, 2004; Mitchell et al., 2004; Chong and Blake, 2006; Kamphuisen et al., 2007), sensory memory (Pearson and Brascamp, 2008), or imagery (Pearson et al., 2008), can bring about a slight imbalance in the neural states, creating a bias that helps one pattern win the race for dominance at the expense of the other.

We have previously demonstrated that imagery can alter future competitive visual interactions in favor of the imagined stimulus on a large percentage of trials (Pearson et al., 2008, 2011), while catch-trial presentations of mock rivalry stimuli do not reveal such bias, ruling out the possibility of demand characteristics (Pylyshyn, 2003). Indeed, scores on offline imagery questionnaires predict imagery strength measured using rivalry (Pearson et al., 2011) and rivalry has been utilized to examine the role of imagery during visual working memory (Keogh and Pearson, 2011). Thus, there is compelling evidence that rivalry bias (or “perceptual bias”) is a useful way to measure imagery strength in general (e.g., encompassing perceptual elements and sensations of vividness). In the current study, imagery strength is the underlying construct of interest, and the extent to which imagery biases perception is taken as a reliable measure of imagery strength. The subjective experiences associated with imagery strength are probed by having participants report the “vividness” of their mental images.

Can people evaluate the phenomenal qualities of internally generated experiences, such as whether a mental image is vivid or detailed? Recently, an attempt was made to answer the question of knowing ones own thoughts (exemplifying the problem of “metacognition;” Flavell, 1979) in relation to mental imagery (Pearson et al., 2011). This study provided compelling new evidence that people have accurate metacognitive knowledge at fine-grained scale, regarding specific instances of imagery: On individual trials, higher ratings of imagery vividness predicted a greater likelihood that the imagined pattern would appear dominant during subsequent rivalry (Pearson et al., 2011). Interestingly, repeated attempts to form a particular visual image can lead to different degrees of success with each try, causing imagery strength to fluctuate from one moment to the next. Despite this variance in imagery strength, people demonstrate good metacognitive understanding of their imagery, and can readily evaluate how vivid their mental images are on a particular occasion.

At a general level, there has been a growing interest in metacognitive judgments of memory and sensory decision-making (Kiani and Shadlen, 2009; Fleming et al., 2010; Rounis et al., 2010; Song et al., 2011). Frontal brain regions are important for introspective or metacognitive ability (Kepecs et al., 2008; Fleming et al., 2010), which suggests that the neural substrates of metacognitive ability are distinct from those supporting primary perception. Although the ability to introspect varies substantially across individuals, within a single individual metacognitive ability seems to be stable and task independent, suggesting a common cognitive process (Song et al., 2011).

Little is known regarding the stability and independence of metacognition of mental imagery. If metacognition for perceptual tasks originates from a common cognitive process, might a similar process allow people to have metacognition of mental imagery? Despite the highly subjective and volitional nature of imagery, people are reasonably good at imagery metacognition (Pearson et al., 2011). Is this ability stable, or might metacognition of imagery improve with repeated practice? Here, we also investigated the degree of imagery metacognition as a function of daily training.

To assess metacognition we use a method derived from signal detection theory (Swets, 1986; Macmillan and Creelman, 1991; Galvin et al., 2003; Kornbrot, 2006) that has been successfully employed in a variety of recent metacognition studies (Fleming et al., 2010; Song et al., 2011). Using this method, we looked at the likelihood that imagery biased subsequent rivalry, given a certain level of imagery vividness. Signal detection allows us to estimate a single quantitative “sensitivity” measure of metacognitive ability, derived from these objective (amount of perceptual bias) and subjective (ratings of vividness) variables. This measure of sensitivity is criterion free, which means that it is not prone to changes in criterion (rating-magnitude), and it is not affected by irregular use of the rating scale (which generally results in unequal numbers of observations across the various conditions).

By way of preview, here we report that imagery strength – measured as the extent to which imagery biases perception during binocular rivalry – did not increase over the 5-day training period. Interestingly, participant’s metacognition of imagery did significantly improve over the training period. This dissociation between imagery strength and metacognitive ability suggests a degree of independence between the two processes.

## Materials and Methods

### Participants

Nine observers (six female) participated in the experiment. All had normal or corrected-to-normal visual acuity and normal stereovision, and all provided written informed consent. Observers received payment for their participation ($10 per hour, plus a $5 per hour bonus upon completion) with the exception of a participating author (RR) and participant BW. The study was carried out with the approval of the Institutional Review Board at Vanderbilt University.

### Materials

Observers viewed the stimuli on a luminance-calibrated CRT monitor with 1152 × 870 resolution and a 75-Hz refresh rate in an otherwise darkened room. Visual stimuli were generated with Matlab 7.5.0 (R2007b) and the Psychophysics toolbox (Brainard, 1997; Pelli, 1997) under Mac OSX. Observers sat at a viewing distance of 56 cm, and used a chinrest to maintain a stable head position. A mirror stereoscope was used to present a different pattern to each eye, and binocular convergence of the two images was aided by a white bull’s eye fixation dot (0.95°) at the center of each monocular half-image. Participants were instructed to maintain steady fixation throughout all experimental trials.

Rivalry stimuli consisted of a green and a red grating (spatial frequency = 1.23 c/°) surrounding a central fixation point, presented against a black background with a mean luminance of 0.09 cd/m^2^. CIE color values of the stimuli were as follows – green: *x *= 0.293, *y *= 0.572; red: *x *= 0.602, *y *= 0.353. Gratings were presented at 75% contrast and had a Gaussian-shaped luminance profile (mean luminance = 6.95 cd/m^2^) that faded to black at the stimulus edge (Gaussian σ = 4.29°). Five observers were trained with a green grating of orientation 112.5° and a red grating of orientation 22.5°, while on generalization blocks they were presented with 67.5° green, and 157.5° red gratings. The opposite was true for the remaining four observers, meaning that we counterbalanced which grating-pairs were used for training and generalization between participants. On catch trials, a mock rivalry stimulus was presented consisting of a physical blend of the green and red rivalry patterns. This stimulus was presented to both eyes simultaneously in order to avoid interocular competition. Presentation of the mock-stimulus allowed us to test for decisional bias and demand characteristics (Landsberger, 1958).

The dominant eye plays a key role in determining which of two monocular images is likely to be perceived at the onset of binocular rivalry. Therefore, individual fine-tuning of stimulus contrast was done before the start of the experiment, and before each daily session, to control for differences in ocular dominance between observers. We used the same procedure as in previous research (Pearson et al., 2008, 2011; Keogh and Pearson, 2011), matching the relative strength of the rivalry gratings to the point at which perceptual competition is most balanced, and thus most susceptible to disruption.

### Procedure

To investigate whether visual imagery can be improved by means of training, and to see how this relates to metacognition of imagery over time, we had observers perform a visual imagery task on five consecutive days, for about an hour a day. A sixth follow-up session was conducted 2–3 weeks after training. Participants came into the lab at or around the same time on each day of training, and were dark-adapted for a couple of minutes before the start of each experimental session.

**Figure 1:**
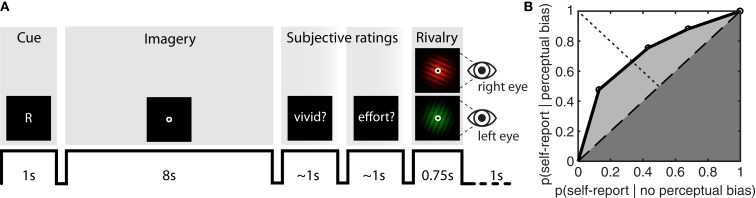
**Sample trial sequence and example ROC II curve. (A)** Participants were presented with a random cue (“G” or “R”) after which they formed a mental image of the green or red grating over an 8-s period. Participants were then cued to report the vividness of the imagined item, and the effort exerted while imagining the item, on an absolute scale from 1 to 4. After a brief flash of the rivalry display, participants reported which grating had appeared perceptually dominant, or whether their percept was an equal mix of the two. On 10% of the trials – instead of the rivalry display – a mock-stimulus was presented to both eyes simultaneously, consisting of a physical combination of both the green and red grating. **(B)** To determine how well subjective ratings predict perceptual bias, type II ROC sensitivity was calculated by taking the area under the ROC curve (*A*_roc_). This area is the sum of the area of the half-square triangle (dark-gray shaded region) and the area between the diagonal and the ROC function (light-gray shaded region).

During the experiment, participants were briefly presented with a randomly chosen (equal number of both) central cue (“G” for green, or “R” for red) at the beginning of each imagery-trial (Figure 1A). Subsequently, participants would engage in visual imagery of the cued pattern for an 8-s period. After completing this imagery period, the word “vividness?” cued participants to first report the quality of their imagery by means of left-handed button presses (1 = *almost no imagery*, 2 = *some weak imagery*, 3 = *moderate imagery*, 4 = *strong imagery almost like perception*), after which they were cued by the word “effort?” to report the amount of vigor with which they had tried to imagine the pattern (1 = *almost no effort*, 2 = *some effort*, 3 = *moderate effort*, 4 = *tried very hard to form a mental image*). Observers were instructed to use the full range of the rating scale to the best of their abilities.

As soon as a participant had responded to both questions, a rivalry display (90% of trials) or a mock display (10% of trials) was presented for 750 ms. On rivalry trials, the green grating was presented to the left eye, and the red grating to the right eye. On mock trials, the plaid-stimulus was presented to both eyes simultaneously. Participants reported which image had appeared most dominant, by pressing one of three buttons (1 = *green*, 2 = *mixed*, 3 = *red*). For this response, the right hand was used in order to minimize potential response conflict between the two hands. A “mixed” response could be made on all trials (rivalry and mock trials). On rivalry trials, the observer could give a mixed response in case he or she was unable to distinguish which grating had appeared more dominant due to binocular combination or piecemeal rivalry. This type of mixed percept was reported on 6.49% of rivalry trials (SEM = 2.49%).

A single training session consisted of two blocks of 70 trials each. Within each block, seven catch trials were randomly interleaved between the rivalry trials. We tested potential generalization of learning to non-trained orientations on day 1 and 5 of training, and during follow-up. On these days, observers performed twice the amount of trials, with training and generalization blocks presented separately and in a randomized order.

### Analyses

**Figure 2:**
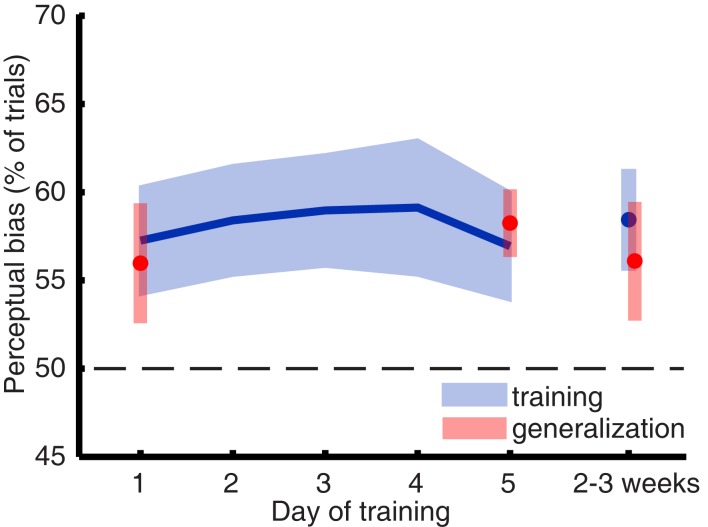
**Imagery biased perception during rivalry in favor of the previously imagined grating, but this perceptual bias did not significantly change over the course of a 5-day training**. The blue line represents the mean training data, and the blue shaded regions represent ±1 SEM. The parts of the plot depicted in red represent data from blocks where we tested training generalization to other orientations.

To assess the strength of visual imagery, we looked at the perceptual facilitation (or bias) of imagery on rivalry. This was calculated as the percentage of trials in which the imagined grating matched subsequent perception during rivalry (Pearson et al., 2008), excluding trials on which a mixed percept was reported. A perceptual bias greater than 50% (chance) on the rivalry trials but not on the catch trials suggests facilitation due to imagery content. Due to experimenter error, a small number of runs (7 out of 108) were missing from the data. Where necessary, we used tri-linear interpolation to infer the mean percentage of bias. For the day-by-day analysis (Figure 2) only one data point was interpolated (percentage perceptual bias for participant CB on day 4); the session-by-session analysis of the same data required interpolation of all seven missing runs.

Data obtained from subjective ratings of vividness (and effort) were analyzed by constructing type II receiver operating characteristic (ROC) curves for each participant on each day of training. This method of assessing metacognitive ability is derived from signal detection methods (Swets, 1986; Macmillan and Creelman, 1991; Galvin et al., 2003; Kornbrot, 2006) and has been successfully employed in a variety of recent inquiries about metacognition (Fleming et al., 2010; Song et al., 2011). Essentially, the only difference between type I and type II tasks lies in the event that is being discriminated: Type I decisions are about the occurrence of events independent of the observer (so, distinguishing between signal and noise), whereas type II decisions are about whether a decision was correct or incorrect (so, making a metacognitive judgment).

Since it is not possible to be “correct” or “incorrect” about an internally generated image, we adapted the definition of the type II decision to include judgments about the vividness of single episodes of mental imagery and its effect on the perceptual outcome during brief instances of subsequent binocular rivalry. Applying the signal detection logic, we categorized trials where participants reported high vividness and where imagery subsequently biased perception as “hits.” Trials where participants reported high vividness but perception was not subsequently biased were categorized as “false alarms.” Here, the ROC II characterizes the probability of a participant being perceptually biased during rivalry, given a certain level of self-reported vividness.

To construct the ROC II curves, we calculated *p*(rating = *i* | perceptual bias) and *p*(rating = *i* | no perceptual bias) for all *i*, and transformed these into cumulative probabilities before plotting them against each other (anchored at [0,0] and [1,1]). Distribution-free methods were employed to characterize type II ROC sensitivity by calculating the area under the ROC curve (*A*_roc_), and type II ROC bias (*B*_roc_). These parameters are derived from simple geometry and do not make assumptions about the shape of the distribution (Kornbrot, 2006). The area under the ROC curve (*A*_roc_) quantifies the extent to which metacognitive judgments are predictive of perceptual bias during rivalry (Figure 1B); a diagonally flat ROC function indicates little predictive value of the metacognitive judgment on the subsequent perceptual outcome during rivalry. The area under the ROC curve is the sum of the area of the half-square triangle (dark-gray shaded region in Figure 1B) and the area between the diagonal and the ROC function (light-gray shaded region in Figure 1B):

(1) $$A_{\text{roc}}=0.25\overset{4}{\underset{i=1}{\sum }}\left[{\left(h_{i+1}-f_{i}\right)}^{2}-{\left(h_{i}-f_{i+1}\right)}^{2}\right]+0.5$$

The bias of the ROC II curve (*B*_roc_) was defined as the ratio *K*_B_*/K*_A_, where *K*_B_ is the area between the ROC curve and the major diagonal (dashed line in Figure 1B) to the right of the minor diagonal (dotted line in Figure 1B), and *K*_A_ is the area between the ROC curve and the major diagonal to the left of the minor diagonal. A neutral bias would give *B*_roc_ equal to zero, while a negative or positive *B*_roc_ indicates a bias toward lower or higher ratings respectively.

(2) $$B_{\text{roc}}=\ln\left(\frac{0.25\overset{2}{\underset{i=1}{\sum }}\left[{\left(h_{i+1}-f_{i}\right)}^{2}-{\left(h_{i}-f_{i+1}\right)}^{2}\right]}{0.25\overset{4}{\underset{i=3}{\sum }}\left[{\left(h_{i+1}-f_{i}\right)}^{2}-{\left(h_{i}-f_{i+1}\right)}^{2}\right]}\right)$$

Previous research has shown that subjective ratings of vividness – but not effort – are predictive of how much perceptual bias someone experiences (Pearson et al., 2011). To confirm this, we also applied the ROC II methods described above to participant’s ratings of exerted effort. This effort-based ROC thus characterizes the probability of a participant being perceptually biased given a certain level of self-reported effort. Finally, to determine whether the ROC II model did a good job accounting for our metacognitive data, we fit a linear regression model:

(3) $$z\left(h\right)=\beta_{0}+\beta_{1}z\left(f\right)+\varepsilon$$

Where *z* is the inverse of the cumulative normal distribution function. The ROC II model provided a good fit to the self-reported vividness (mean *R*^2^ = 0.976 ± 0.004) and effort data (mean *R*^2^ = 0.981 ± 0.007).

## Results

### Imagery training

Sustained mental imagery can bias the perception of an ambiguous display, resulting in a reliable measure of imagery strength on a trial-to-trial basis (Pearson et al., 2008, 2011). When people rate their imagery as more vivid, the likelihood that imagery influences perception is larger (Pearson et al., 2011). Thus, if training mental imagery would result in more vivid images, one would expect to see an increase of perceptual bias over time. Figure 2 shows the mean imagery strength (or “perceptual bias”) as a function of days of training and again 2 weeks later. A within-subjects ANOVA revealed that training did not increase the amount of perceptual bias over time [*F*_(5,40)_ < 1].

Mental imagery did bias perception in favor of the imagined grating [*F*_(5,40)_ = 8.861; *p* = 0.018] which is consistent with previous work demonstrating the effect of mental imagery on rivalry (Pearson et al., 2008, 2011). Unsurprisingly – considering the lack of a training effect – gratings of both trained and untrained (generalization) orientations yielded similar perceptual biases: a within-subjects ANOVA for training days 1, 5, and follow-up revealed no main effect of orientation [*F*_(1,8)_ < 1]. Analyzing the data by session did not unveil any hidden differences in perceptual bias over time [*F*_(11,88)_ = 1.106; *p* = 0.366], which excludes the possibility that most learning took place between the first couple of sessions.

Additional evidence that mental imagery was not improved by training comes from participant’s introspective judgments of imagery vividness. Mean self-reported vividness of mental imagery was statistically the same on all days of training [*F*_(5,40)_ = 1.224; *p* = 0.316]. Self-reports of exerted effort did not change over the course of training either [*F*_(5,40)_ < 1]. In summary, neither the perceptual measure of imagery strength (“perceptual bias”) nor ratings of vividness showed any significant change over the 5-days of training. Thus, it appears that training in this study was unable to increase imagery strength over time.

### Catch trials

**Figure 3:**
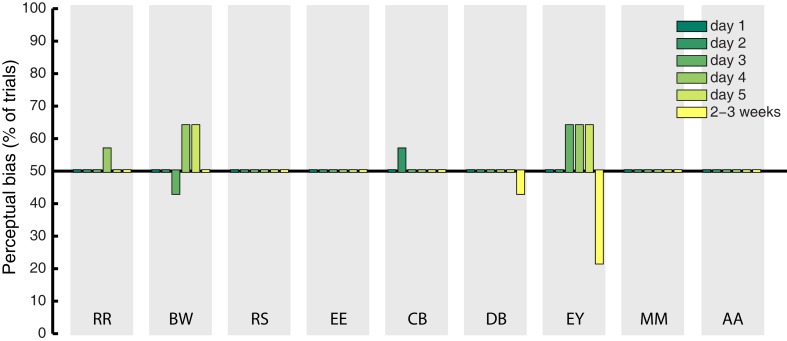
**Mean percentage of catch trials on which participant’s response to the fake-rivalry display was biased in favor of the imagined grating**. A score of 50% indicates a lack of bias. Gray shaded areas show each individual participant, the colored bars represent the 5 days of training plus follow-up 2–3 weeks later.

Catch trials were presented in a randomly interleaved fashion on 10% of all experimental trials, to determine whether observers showed response bias in favor of the imagined grating. On these trials, a mock rivalry display was presented consisting of a balanced physical combination of the green and red gratings shown to both eyes simultaneously. If the effects observed during rivalry were due to decisional bias or demand characteristics, we expect to find the same degree of response bias on catch trials. We analyzed bias by coding veridical “mixed” responses to the catch trials as 50%, while responses that matched the cued pattern were coded as 100%, and responses opposite to the cued grating were coded as 0%. The percentage of catch trials during which participant’s responses were biased in favor of the cued grating are shown in Figure 3 (for all days of training). On average, this bias was 50.79%. This indicates that demand characteristics and decisional bias have a negligible influence on participant’s reports of rivalry dominance, as previously documented (Pearson et al., 2011).

### Metacognitive judgments

**Figure 4:**
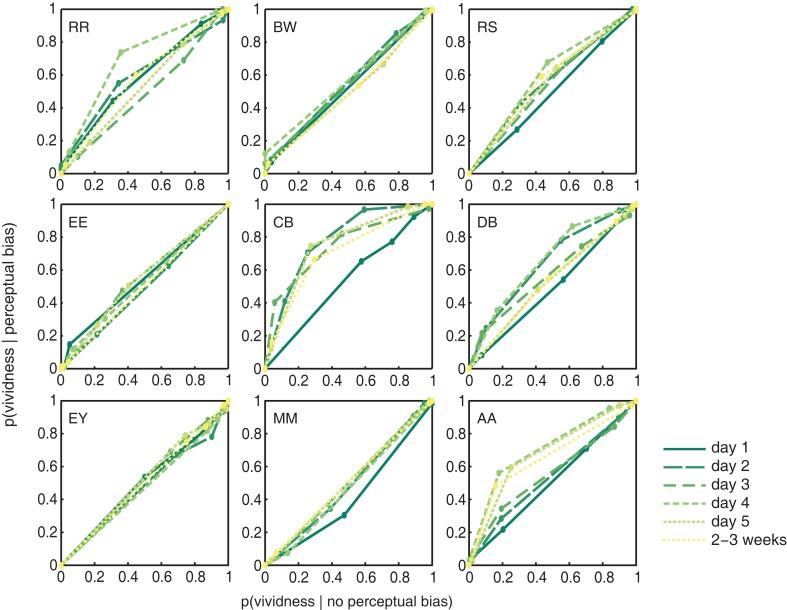
**Participant’s metacognition of imagery over the course of training**. ROC II curves based on the vividness ratings reported by our participants (collapsed over Gabor orientations). Each plot represents an individual participant; the colored lines represent the ROC curves for the different days of training.

To assess whether people’s metacognitive insights about imagery strength improve over the course of training, we constructed ROC II curves for each individual observer, on each day of training (Figure 4; Materials and Methods). The extent to which metacognitive judgments of vividness predict perceptual bias was quantified as the area under each ROC II curve. Data presented in Figure 4 demonstrate that on earlier days of training (darker green lines) the area under the curve is smaller than on later days of training (lighter green lines). The upward bowing profile of the curves observable in over half of our participants demonstrates that vividness judgments indeed predict perceptual bias.

There is a clear trend toward more metacognitive ability over time for both the trained [*F*_(5,40)_ = 1.742; *p* = 0.147] and untrained (generalization) orientation [*F*_(2,16)_ = 7.416; *p* = 0.005]. Trained and untrained orientations do not statistically differ [*F*_(1,8)_ < 1]. This lack of orientation specificity may not be surprising considering that metacognition for perception is something presumably supported by higher-level frontal areas of the brain (Fleming et al., 2010), where responses are invariant to fine-grained orientation information. Hence, we simplified our analysis by collapsing the data from all orientations before constructing the ROC II curves as displayed in Figure 4. Estimates of the type II ROC sensitivity *A*_roc_ are therefore slightly more reliable on day 1, day 5, and during follow-up, since they are constructed based on more data.

**Figure 5:**
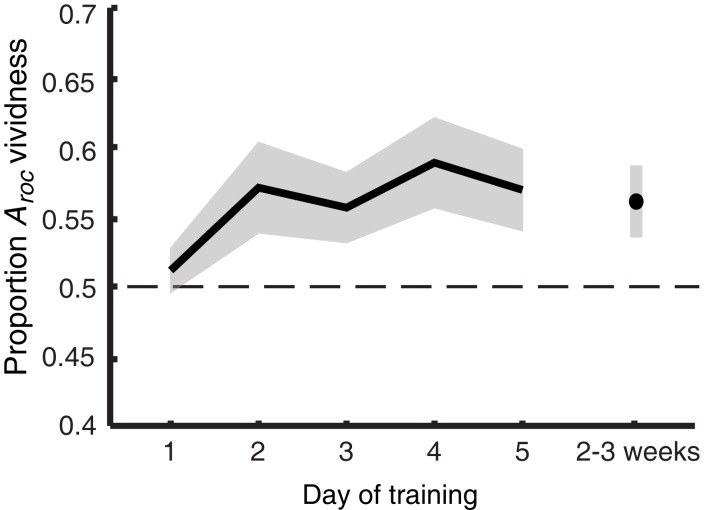
**Imagery metacognition improves over the course of training**. The extent to which self-reported vividness predicts perceptual bias (quantified as the area under the ROC II curve after collapsing all grating orientations, as also shown in Figure 4) plotted against day of training. Shaded areas represent ±1 SEM.

The information from Figure 4 is summarized in Figure 5, showing the main effect of training: vividness judgments predict perceptual bias increasingly better over time [*F*_(5,40)_ = 3.075; *p* = 0.019]. This trend is linear when only looking at training days 1–5 [*F*_(1,8)_ = 5.846; *p* = 0.042] but becomes quadratic when follow-up is included [*F*_(1,8)_ = 8.778; *p* = 0.018], indicating a drop of the proportion *A*_roc_ at follow-up. Nevertheless, planned comparisons (uncorrected *t*-tests) show that – with the exception of day 1 – the predictive value of self-reported vividness on the perceptual outcome is larger than would be expected by chance (one-tailed one-sample *t*-test day 1: *p* = 0.243; all others: *p* < 0.029), and this ability is still present 2–3 weeks after training (*p* = 0.021).

In previous work we demonstrated that people can reliably evaluate the vividness of their mental imagery from one trial to the next (Pearson et al., 2011). Participants in that previous study were tested only once. Thus, one might expect to find evidence for metacognition of imagery strength on day 1 of training in the current experiment. However, the difference in analyses used to determine metacognition in our previous and current work (within-subjects analysis of variance, and area under ROC II curve respectively), make it hard to directly compare the findings. A within-subjects analysis of variance performed on the current data shows that on day 1 of training, participants marginally (but not significantly) showed a main effect of vividness on perceptual bias [*F*_(3,15)_ = 2.83; *p* = 0.074]. However, a lack of observed power (0.558) indicates that at this sample size there is only a small (44%) chance of finding a significant effect (at α = 0.05) when assuming that people have metacognitive insights into their own imagery strength at the population level. An *a priori* power analysis indicates that, assuming a medium effect size, 21 subjects would be required to obtain a power of 0.95.

**Figure 6:**
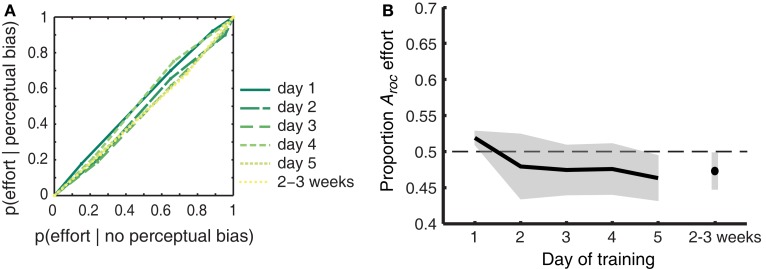
**Participant’s self-reported effort with which the gratings were imagined does not predict perceptual bias**. **(A)** ROC II curves based on the effort ratings pooled across participants. Green colored lines represent the different days of training. The diagonally flat functions indicate a weak link between self-reported effort ratings and perceptual bias during rivalry. **(B)** The extent to which self-reported effort predicts perceptual bias (quantified as the area under the ROC II curve) plotted against each day of training. Self-reported effort does not predict perceptual bias, and this does not change over time. Data was collapsed over Gabor orientations, since outcomes did not differ between the two sets of orientations [*F*_(1,8)_ < 1]. Shaded areas represent ±1 SEM.

Vividness ratings are predictive of the efficacy that mental imagery has at biasing the perception of rivaling stimuli. By contrast, self-reported effort for imagery was not hypothesized to predict perceptual bias. Attempts to exert greater effort do not necessarily result in highly effective imagery, as demonstrated by previous work (Pearson et al., 2011). To ensure that our findings were specific to introspective vividness – and not effort – we constructed ROC II curves (as in Figure 4; Materials and Methods) based on the effort ratings reported by our participants. The pooled (across participants) curves per day are shown in Figure 6A; the diagonally flat function indicates a weak link between self-reported effort ratings and perceptual bias during rivalry. Figure 6B demonstrates that, as expected, effort did not predict perceptual bias [*F*_(1,8)_ < 1]. The area under the ROC II curve (*A*_roc_), which quantifies the degree to which self-reported effort predicts perceptual bias during rivalry, did not differ from chance on any of the training days (two-tailed one-sample *t*-tests all *p* > 0.081). Neither did we observe a change over time for the trained [*F*_(5,40)_ < 1], untrained (generalized) [*F*_(2,16)_ = 2.711; *p* = 0.097], or collapsed [*F*_(5,40)_ < 1] grating orientations.

Vividness appears to predict perceptual bias more strongly over the course of training, whereas effort does not predict perceptual bias at all. Can this finding be explained by the way participants used the rating scales? Participant’s average reported vividness (2.57 ± 0.21) and effort (2.86 ± 0.14) did not significantly differ (*p* = 0.261). In other words, subjective vividness – but not effort – is predictive of how well something was imagined independent of rating-magnitude. Signal detection theory considers metacognitive ability (sensitivity) and rating-magnitude (bias) as two independent properties (Galvin et al., 2003). In accordance with this notion, we find that individuals with higher self-reported vividness were not better at evaluating their imagery strength and vice versa. Specifically, participant’s ability to make accurate metacognitive judgments of their mental imagery (*A*_roc_ vividness) and participant’s mean vividness ratings were not correlated (*r* = 0.063; *p* = 0.873).

The type II bias of the ROC curve (*B*_roc_) provides us with a distribution-free estimate of the criterion used by participants to provide their subjective ratings. A neutral bias would give *B*_roc_ equal to zero, while a negative or positive *B*_roc_ indicates a bias toward lower or higher ratings respectively. This estimate corresponded very well with the actual rating-magnitude collected during the experiment: self-reported vividness ratings and estimated vividness bias (*B*_roc_ vividness) were highly correlated (*r* = 0.913; *p* < 0.001), as were self-reported effort and estimated effort bias (*r* = 0.889; *p* = 0.001). The close resemblance between these two variables – both measures of participant’s criterion – helps validate the distribution-free approach used to determine ROC estimates in the current paradigm.

Strikingly, Figure 4 shows large differences between individuals: the degree to which metacognitive vividness judgments predict perceptual bias varies quite a bit from one person to the next (*A*_roc _= 0.48–0.71). This type of variability is not uncommon, and previous studies have reported similarly large individual differences in metacognitive ability for perceptual tasks (Song et al., 2011). Besides large differences related to metacognition, also the overall amount of perceptual bias experienced by our participants varied widely (46–76%). Nonetheless, participant’s overall metacognitive ability and the percent perceptual bias they experienced throughout the experiment, were uncorrelated (*r* = −0.027; *p* = 0.945). This suggests that participant’s metacognitive ability in this task is independent of imagery strength, as measured by binocular rivalry.

## Discussion

The research presented here suggests that mental imagery strength does not improve over the course of our 5-day training regime. Over the 5-days, no changes were observed relating to imagery strength as measured by rivalry, nor were there any changes in the average introspective judgments of imagery vividness. We further demonstrated that self-reported vividness of mental imagery predicts the perceptual consequences of single epochs of imagery. More importantly, this prediction becomes stronger with practice, implying increased metacognition of imagery over the course of training. Self-reported effort of mental imagery on the other hand, did not predict perceptual outcomes.

There have been reports of visual imagery increasing performance on subsequent perceptual tasks (Tartaglia et al., 2009). Yet we were unable to find an increase in facilitation of rivalry dominance after 5 days of training. The question is of course, why? The emphasis of the research presented here was on improving imagery strength over time. This is a notably different emphasis from studies that have investigated how imagery training changes perceptual skills (Tartaglia et al., 2009). One obvious explanation for the lack of an imagery training effect in this study is that imagery strength simply cannot improve with practice. This idea is corroborated by the fact that neither imagery bias, nor subjective ratings of imagery strength showed a significant increase as a function of training. Introspective ratings of imagery strength are reflected in the perceptual outcomes during rivalry, and the close relationship between the two implies they measure the same underlying construct (Pearson et al., 2011). Thus, several aspects of the data support the hypothesis that it is not possible – or very difficult – to improve imagery strength by means of training.

The idea that training cannot easily change imagery strength might be explained by the manner in which imagery strength is linked to brain anatomy. The Tartaglia study (Tartaglia et al., 2009) had participants repetitively imagine the crucial part of a bisection stimulus (spatial judgment) or a low-contrast Gabor pattern (contrast judgment). They found improved perceptual performance on a subsequent perceptual bisection task and a Gabor detection task after imagery training, and this improvement generalized to untrained orientations. This lack of orientation specificity implies that learning through imagery did not involve plastic changes in early visual cortex, but probably involved higher-level extra-striate areas. Higher-level changes may boost perceptual performance through imagery training, yet, changes at this cortical level may not be sufficient to improve imagery strength itself.

Historically, mental imagery has been considered a fainter form of perception (Hume, 1739). Evidence to support this notion comes from functional magnetic resonance imaging (fMRI) studies demonstrating that the magnitude of brain activity is lower during imagery than during bottom-up perception (Goebel et al., 1998; O’Craven and Kanwisher, 2000). Likewise, single neuron recordings in the medial temporal lobe of humans found fewer neurons that were recruited during imagery than during perception, and that the firing rate of these cells was lower during imagery compared to perception (Kreiman et al., 2000). In the case of perceptual bias during rivalry, imagery is presumed to influence or boost the memory trace that exists between one rivalry presentation and the next, and the location and orientation specificity of this memory trace implies that it is composed of primarily low-level characteristics (Ishai and Sagi, 1995; Pearson et al., 2008; Slotnick, 2008). Mechanisms such as a gain in sensitivity for the imagined pattern, or the strengthening of sensory traces, would be needed to modify population activity in lower visual areas mediating alternations of conscious perception during rivalry. Imagery may simply lack sufficient impact to induce permanent plastic changes at these lowest sensory levels. Future research directions aiming to improve imagery strength could investigate the necessity of bottom-up information for learning. Specifically, it would be interesting to see if there is a transfer from improving visual perception by means of prolonged training with actual sensory stimuli, to improvements of imagery strength.

One could hypothesize that imagery strength is liable to improvement, but we simply failed to find any in this study due to the configuration of our task. Research into the process of improving perceptual skill – or perceptual learning – provides useful context in support of this hypothesis. One influential view known as the reverse hierarchy theory (Ahissar and Hochstein, 2004), states that learning is gated by top-down, task-related factors: Learning begins at high-level areas of the brain, after which it trickles down the hierarchy, fine-tuning the read out from lower level areas. This theory invokes a number of detailed predictions, namely, early (fast) learning should be related to high-level changes, whereas asymptotic (slow) learning should involve plasticity in low-level sensory areas – if required by the task. There is considerable evidence supporting this view (Ahissar and Hochstein, 1993, 1997; Dosher and Lu, 1998; Dupuis-Roy and Gosselin, 2007).

In light of the reverse hierarchy framework, our training regime is suspect to a critical vulnerability. Namely: training duration. Five days may have been insufficient time to reach the asymptotic learning phase. The Tartaglia study previously mentioned (Tartaglia et al., 2009) trained participant’s imagery for 10 days, twice as long as in our study, and found an improvement on perceptual tasks. Assuming that specific cellular plastic processes at the hierarchical level of ocular dominance columns can only occur during asymptotic learning, longer training might be necessary when aiming to influence rivalry perception.

Recent research has demonstrated that perceptual learning can also occur without a specific task and outside of awareness, as long as the information of interest is paired with feedback or a reward signal (Seitz and Watanabe, 2003, 2005; Seitz et al., 2009) or with online-feedback via decoded fMRI signals (Shibata et al., 2011). Our experimental design lacked a direct reward signal. Perhaps if successful epochs of imagery were paired with a reward signal, this could facilitate learning. In practice the implementation of a reward may prove difficult to realize. Often, measures of imagery strength are dependent on subjective reports, and offering rewards based on only self-reports could induce strong response and observer biases. Nevertheless, it is possible that our training was insufficient to obtain an effect, and providing feedback, rewards, or some manner of getting participants to intentionally try and increase their imagery strength, could have been a more effective way to train mental imagery.

During memory consolidation, initially fragile memory traces become stabilized due to practice-induced plasticity in task relevant brain areas (Karni, 1996; Dudai, 2004). Can the ineffectiveness of imagery training be due to somehow disrupted memory consolidation? Classically, consolidation has been defined as a time limited process directly following learning (Dudai, 2004). However, recent studies indicate that interference is rather time independent, and can occur at long intervals after training (Goedert and Willingham, 2002; Caithness et al., 2004; Zhang et al., 2008). Interference can be considered strongly stimulus dependent, resulting from similarity between the learned and interfering stimulus, and the corresponding neuronal populations recruited by these stimuli (Seitz et al., 2005; Been et al., 2011). Specifically, for Gabor patterns most interference occurs when interfering stimuli differ from the learned orientation by 30°, while no interference is observed from orthogonal orientations (Been et al., 2011). Considering the orthogonal training orientations of our experiment, disruption of consolidation seems an unlikely explanation for the ineffectiveness of imagery training.

Can people become better at knowing their own thoughts? We were able to improve subject’s ability to judge the vividness of their imagery. This improvement was still present during a follow-up test, implying a long lasting effect of training on metacognitive evaluation of mental imagery. Furthermore, training of metacognition was not orientation specific: metacognition was improved for both trained and untrained sets of orientations. It is likely that the improvement of metacognition reported here originates from higher-level brain areas. This is in concordance with the suspected high-level neural locus of metacognitive ability for perception (Kepecs et al., 2008; Kiani and Shadlen, 2009; Fleming et al., 2010) as well as the idea that networks in high-level cortical regions orchestrate strategic choices during early learning, allocating attention and motivation in response to specific task demands (Willingham, 1999; Hochstein and Ahissar, 2002; Doyon et al., 2003).

Our study demonstrated improvements of the metacognition of imagery, whereas no changes in imagery strength itself were observed. This dissociation suggests distinct brain mechanisms underlying metacognition and visual imagery respectively. Similar distinctions have been made regarding metacognition of perception: Neuroanatomical substrates of introspective ability are distinct from those supporting primary perception (Fleming et al., 2010), and there is a marked dissociation between metacognitive ability and performance on visual perceptual tasks (Lau and Passingham, 2006; Lau, 2008; Rounis et al., 2010; Song et al., 2011). Thus, metacognitive ability can be viewed as a stable and task independent cognitive process that can be improved with practice, independent of performance on other tasks. Changes in high-level neuronal populations are likely candidates for this learning.

The ability to introspect on private thoughts is key to human subjective experience. Yet, people’s ability to evaluate internally generated experiences – such as imagery – is not as self-evident as it may appear. Although a large number of studies now demonstrate that something as private as a mental image can be successfully studied from a third-person perspective (Ishai and Sagi, 1995, 1997; Kosslyn et al., 2001; Pearson et al., 2008; Tartaglia et al., 2009), research has only recently begun to tackle issues related to the first-person perspective (Pearson et al., 2011). The core problem from the first-person perspective of the imaginer is that self-generated instances of imagery, unlike perception, cannot be directly compared with a perceptual template. Nevertheless, people seem quite capable of knowing if a mental image is accurate, vivid, or detailed. And practice further improves this first-person introspective ability. Why might such metacognitive knowledge be important?

Introspective or “metacognitive” sensitivity is important to guide actions and to make decisions (Vickers, 1979; Daw et al., 2005; Dayan and Daw, 2008) and being able to adequately estimate ones confidence can help drive adaptive behavior (Kepecs et al., 2008). In its simplest form, low confidence that a recent decision was correct may prompt reexamination of the evidence, or seeking a second-opinion. In the event of internally generated experiences such as mental imagery, low confidence that an image was veridical and life-like may lead someone to reconsider such an experience. A better metacognitive understanding may help the imaginer bridge the gap between first and third-person perspective. For example, people can resolve potential ambiguities about perception by comparing their own perceptual experience with the subjective experience of another person (Bahrami et al., 2010). Similarly, when the imaginer has a better understanding of the authenticity of his or her mental image, it will be easier to communicate its content to another person. In sum, increasing the efficiency with which people introspect the quality of their mental images can prove a novel and important finding.

In conclusion, we discussed a variety of reasons why training did not lead to an improvement of imagery strength in the current study. Such an improvement may simply be very difficult to document, or our task may not have been optimally suited to detect improvements of imagery strength. Nevertheless, we demonstrated that people’s ability to introspect their own imagery strength does improve with training, which suggests distinct mechanisms underlying imagery and metacognition. Being able to improve metacognition by means of practice can have important implications for real-life situations. It would be interesting to know if training metacognition could help people improve certain cognitive functions, such as decision-making or planning actions. If so, this may prove especially helpful for specific patient populations. Finally, future investigations of prolonged training of imagery can prove advantageous in outlining the overlap between mechanisms of perception and imagery. Imagery as defined here is a highly voluntary process that allows introspection in the absence of direct perceptual input. As such, imagery can provide a unique gateway to understanding how perceptual and introspective processes are represented in the brain.

## Conflict of Interest Statement

The authors declare that the research was conducted in the absence of any commercial or financial relationships that could be construed as a potential conflict of interest.
